# The Patient Insisted upon Under-Going Abdominal Section!

**Published:** 1898-03-12

**Authors:** 


### "THE PATIENT INSISTED UPON UNDERGOING ABDOMINAL SECTION!"

A curious point arose in the discussion on this paper at the Medical Society, which, although it seemed to excite no attention at the time, is, we think, worthy of consideration, if only on account of the questions which are involved in what we may call the ethics of operations. Two cases were related, in both of which it appeared that the patients "insisted on the abdomen being opened." Fortunately in both of them the operation was followed by relief, and in one case by relief far greater than was expected, for not only was the patient, who was in an advanced state of phthisis, relieved of the abdominal condition for which the operation was performed, but her lungs also improved to such an extent that she had since recovered a fair condition of health. Still one can hardly help asking, Where is to be the limit of what a medical man will do at the request of a patient? when we hear of surgeons performing laparotomy because their patients "insist" upon its being done.

